# GPR Clutter Removal Based on Weighted Nuclear Norm Minimization for Nonparallel Cases

**DOI:** 10.3390/s23115078

**Published:** 2023-05-25

**Authors:** Li Liu, Chenyan Song, Zezhou Wu, Hang Xu, Jingxia Li, Bingjie Wang, Jiasu Li

**Affiliations:** 1 Key Laboratory of Advanced Transducers & Intelligent Control System, Ministry of Education and Shanxi Province, Taiyuan University of Technology, Taiyuan 030024, China; liuli01@tyut.edu.cn (L.L.); songchenyan1238@link.tyut.edu.cn (C.S.); zezhouwu97@163.com (Z.W.); wangbingjie@tyut.edu.cn (B.W.); lijiasu1246@link.tyut.edu.cn (J.L.); 2 College of Electronic Information & Optical Engineering, Taiyuan University of Technology, Taiyuan 030024, China

**Keywords:** ground-penetrating radar, clutter removal, low-rank and sparse decomposition, weighted nuclear norm minimization

## Abstract

Ground-penetrating radar (GPR) is an effective geophysical electromagnetic method for underground target detection. However, the target response is usually overwhelmed by strong clutter, thus damaging the detection performance. To account for the nonparallel case of the antennas and the ground surface, a novel GPR clutter-removal method based on weighted nuclear norm minimization (WNNM) is proposed, which decomposes the B-scan image into a low-rank clutter matrix and a sparse target matrix by using a non-convex weighted nuclear norm and assigning different weights to different singular values. The WNNM method’s performance is evaluated using both numerical simulations and experiments with real GPR systems. Comparative analysis with the commonly used state-of-the-art clutter removal methods is also conducted in terms of the peak signal-to-noise ratio (PSNR) and the improvement factor (IF). The visualization and quantitative results demonstrate that the proposed method outperforms the others in the nonparallel case. Moreover, it is about five times faster than the RPCA, which is beneficial for practical applications.

## 1. Introduction

Ground-penetrating radar (GPR) is a non-destructive electromagnetic technique for detecting subsurface targets and has been widely used in many civilian and military applications [1,2,3,4,5,6]. However, underground target detection is usually impaired by the strong clutter caused by the ground reflection, the direct coupling between the transmitting and receiving antennas, and reflections from subsurface discontinuities, yielding a low signal-to-clutter ratio (SCR) for the echo signal. Therefore, clutter removal is an essential preprocessing technology for GPR target detection.

Many clutter removal methods have been proposed. The most common method is mean subtraction (MS) [7], but this spatial filter will affect the intensity of the target response. Other spatial filters, such as median filter and exponential moving average filter (EMA) [8], are also used for clutter removal. Subspace-based methods, such as principal component analysis (PCA) [9,10], independent component analysis (ICA) [10,11], or singular value decomposition (SVD) [12,13], decompose the GPR image into the clutter, target, and noise components and the clutter can be suppressed by removing the most dominant component. However, these methods are not suitable for multiple target cases or shallowly buried target cases since the targets may not be represented by a single component and the clutter component may be interrelated with the target component. Recently, Gaussian curvature decomposition (GCD) in the PCA domain was proposed to extract target information from the clutter and random noise [14]. Morphological component analysis (MCA) [15] based on sparse representations decomposes the image into morphological components, where each component can be sparsely represented by an appropriate dictionary. It requires prior knowledge for dictionary learning and has high computation complexity. Ni et al. [16] combined frequency-wavenumber migration and dictionary learning to separate the focused target response with a point-shaped structure from the clutter with a horizontal trip-shaped structure.

Multi-resolution and multi-direction decomposition methods based on curvelet transform [17,18], a multi-scale directional bilateral and neighborhood filter [19], and a lattice filter [20] are also proposed. Since the clutter has a horizontal direction and the target has a hyperbolic direction, these methods decompose the GPR image into multi-scale subbands, each of which is also decomposed into multiple directional subbands. The clutter can be suppressed by removing the subbands corresponding to the clutter component.

Robust principal component analysis (RPCA) [21], which decomposes the GPR image into a low-rank clutter matrix and a sparse target matrix, has demonstrated its superiority to the conventional subspace-based methods in GPR clutter removal [22,23]. Motivated by its success, various low-rank and sparse decomposition (LRSD) methods are successively proposed [24,25,26,27,28,29,30,31]. Song et al. [24] presented an improved RPCA method that focuses on the target response by migration imaging, followed by suppressing the clutter using the RPCA. Later, they proposed an efficient RPCA-based method, called the Go Decomposition (GoDec) to extract the target image for antipersonnel mine detection. This method uses bilateral random projections rather than the singular value thresholding operator to achieve high computational efficiency [25]. Kumlu et al. [26] applied the nonnegative matrix factorization (NMF) method to GPR clutter removal. Later, they exploited the robust NMF (RNMF) method and validated that it has a higher performance than the RPCA in terms of operation speed and clutter reduction results [27]. However, this method is sensitive to the regularization parameter. They also presented a two-step GoDec approach for cases with missing data [28]. Moreover, they exploited a robust orthonormal subspace learning (ROSL) method for GPR clutter reduction [29]. This method has a faster implementation and comparable performance to GoDec and RNMF without presetting the parameters. In our earlier work, factor group-sparse regularization uses a non-convex matrix factorization as a surrogate for the matrix rank rather than the nuclear norm, as in the RPCA [30]. We also investigated clutter removal based on tensor RPCA using GPR C-scan data [31].

In 2020, Ni et al. [32] introduced a clutter-removal method based on a robust autoencoder, which utilizes RAE to solve the low-rank and sparse matrix representation problem. Later, deep-learning-based clutter-suppression methods were put forward, such as a convolutional autoencoder [33], RNMF-guided deep network [34], generative adversarial nets (GAN)-based methods [35,36], and a clutter-removal neural network with U-net architecture [37], but these methods need a large amount of data to train the network, which is not easy or convenient in the GPR field.

Despite the intensive research on clutter removal, few studies have considered cases where there is an angle between the antennas and the ground surface. In practical applications, especially in pavement inspection, ground-coupled GPR requires the attachment of antenna to the road surface, limiting its operation speed and causing traffic interruptions; therefore, road-detecting vehicles usually use air-coupled antennas, which are installed above the ground to speed the detection process and avoid lane closures. For an air-coupled GPR, when the ground has potholes, when the vehicle is moving across a speed bump, or when the antennas are not exactly parallel to the ground due to an installation error, there may be an inclination angle between the antennas and the ground. In this case, the clutter caused by the ground reflection is more complex, and the existing clutter-removal methods may not be effective enough.

In this paper, we propose a novel GPR clutter-removal method based on weighted nuclear norm minimization (WNNM) [38], focusing on the case when the antenna is not parallel to the ground surface. The WNNM method uses a non-convex weighted nuclear norm surrogate for the rank. It assigns different weights to different singular values, making it more flexible when solving real problems. The contribution of this paper is that we introduce the WNNM into GPR clutter removal to account for the nonparallel case of the antenna and the ground, which occurs in practice but has not been considered in the literature to date.

The rest of the paper is organized as follows. Section 2 introduces the proposed clutter removal algorithm based on the WNNM. The visual and quantitative results for simulated and real data are presented in Section 3. Section 4 finally provides the conclusions drawn from the results.

## 2. Methodology

### 2.1. GPR Clutter Removal via Low-Rank and Sparse Decomposition

Mathematically, a GPR B-scan data matrix **X** can be represented by the sum of a low-rank clutter matrix **L** with a few nonzero singular values, a sparse target matrix **S** with a few nonzero entries, and a noise matrix **N**

(1) $$\;X=L+S+N\;L,S,N\in {\mathbb{R}}^{M\times N}$$

where *M* is the number of time samples in each A-scan trace and *N* is the number of traces. The clutter is caused by the ground reflection, the direct wave (including antenna internal reflections), and the multiple reflections between antennas and the ground. The scattering response from non-targets (e.g., gravel, roots) and other small subsurface discontinuities are included in the noise term. Thus, the clutter removal problem is cast as a low-rank and sparse decomposition optimization problem.

In the RPCA, the problem is solved by a tractable convex optimization [21,39]

(2) $$\;\underset{L,S}{\min}\;{\left\|\;L\;\right\|}_{*}\;+\;\lambda \;\;\left\|\;S\;\right\|{\;}_{1}\;\;\;\;\;s.\;t.\;\;{\left\|\;X-L-S\;\right\|}_{F}\leq \varepsilon$$

where ${\left\|.\right\|}_{*}$ is the nuclear norm defined as the sum of the singular values of the matrix, $\;{\left\|.\right\|}_{1}\;$ refers to the *l*_1_-norm, namely the sum of the absolute values of the matrix entries, and *λ* is a regularization parameter that balances the contribution of the low-rank and the sparse component, and is suggested to be 1/(max(*M*,*N*)^1/2^) in many RPCA applications. $\;{\left\|.\right\|}_{F}\;$ is the Frobenius norm, and *ε* is a positive constant related to the noise level.

### 2.2. Proposed WNNM-Based Clutter Removal Method

The widely used RPCA solves the nuclear norm by iterative SVD and updates **L** using the soft thresholding operator to shrink each singular value by the same parameter. This is not very reasonable since different singular values may have different levels of importance and should be treated differently. In fact, each singular value of GPR raw data has a clear physical meaning. The larger singular value corresponds to the clutter caused by the ground surface and the interfaces in the layered media structures, and the other singular values may correspond to the target. Thus, it is sensible to greatly suppress the larger singular value and retain the smaller singular value as much as possible. When the antenna is not parallel to the ground, the ground clutter no longer presents linear horizontal characteristics. If each singular value of the low rank matrix is treated the same, partial ground clutter may not be effectively removed, whereas if singular values are treated differently, complete ground clutter removal is theoretically possible.

The WNNM just assigns different weights to different singular values, and the optimization problem can be rewritten as

(3) $$\;\underset{L,S}{\min}\;{\left\|\;L\;\right\|}_{w,*}\;+\;\lambda \;\left\|\;S\;\right\|{\;}_{1}+\;\frac{1}{2}\;{\left\|\;X-L-S\;\right\|}_{F}^{2}$$

where $\;{\left\|\;.\;\right\|}_{w,*}$ denotes the weighted nuclear norm of the matrix and can be defined as

(4) $$\;{\left\|\;L\;\right\|}_{w,*}=\;\sum_{j}w_{j}\sigma_{j}(L)\;\;\;\;s.\;t.\;\;w_{j}\geq 0\;$$

where *σ_j_* and *w_j_* are the *j*-th largest singular value of matrix **L** and its assigned weight, respectively. A diagonal weight matrix **W** can be obtained by diag[*w*_1_, *w*_2_, *w*_3_, *… w*_j_, *…*]. Considering that clutter reflections dominate in the GPR data, the larger the singular values, the smaller the weight that should be assigned, and thus **W** can be denoted as

(5) $$\;W=\left[\begin{array}{ccccc} w_{1} & & & & \\ & w_{2} & & & \\ & & \;\ddots \; & & \\ & & & \;\;w_{j}=\frac{\rho }{\sigma_{j}+\tau } & \\ & & & & \ddots \end{array}\right]$$

where *ρ* is a positive weight parameter. *τ* is a small positive constant to avoid dividing by zero.

Equation (2) can be solved by the inexact alternating direction method of multipliers (ADMM) [40]. The alternate minimization steps are

(6) $$\;\left\{\begin{array}{l} L^{t}=\arg\underset{L}{\min}{\left\|L^{t-1}\right\|}_{w,*}\;\;+\;\frac{1}{2}\;{\left\|X-L^{t-1}-S^{t-1}\right\|}_{F}^{2}\\ S^{t}=\arg\underset{S}{\min}\lambda \;\;\left\|\;\;S{\;}^{t-1}\;\right\|{\;}_{1}+\frac{1}{2}\;\;{\left\|X-L^{t}-S^{t-1}\right\|}_{F}^{2} \end{array}\right.$$

where *t* denotes the *t*-th iteration. **L***^t^* and **S***^t^* can be updated by

(7) $$\;\left\{\begin{array}{l} U\Lambda V^{T}=\mathrm{svd}(X-S^{t-1})\\ L^{t}=U\varphi_{W}(\Lambda )V^{T}\\ S^{t}=\varphi_{\lambda }(X-L^{t}) \end{array}\right.$$

where svd(·) denotes the singular value decomposition. **U**, **Λ**, and **V** are the left singular matrix, singular value matrix and right singular matrix obtained by svd(**X** − **S***^t−^*^1^), respectively. *φ*(·) is the element-wise soft thresholding operator, and $\;\varphi_{W}(\Lambda )$ can be presented as

(8) $$\;\varphi_{W}(\Lambda )=\left\{\begin{array}{l} \Lambda_{i,j}-W_{i,j}\;\;\;\;,\mathrm{if}\;\;\;\Lambda_{i,j}\geq W_{i,j}\\ \Lambda_{i,j}+W_{i,j}\;\;\;\;,\mathrm{if}\;\;\;\Lambda_{i,j}<W_{i,j}\\ \;\;0,\;\;\;\;\;\;\mathrm{otherwise} \end{array}\right.$$

where *i* and *j* are pixel locations. When one of the convergence conditions is satisfied or the maximum iteration is reached, the iteration is stopped. The convergence conditions can be represented as

(9) $$\;\frac{{\left\|L^{t}-L^{t-1}\right\|}_{F}^{2}}{{\left\|L^{t}\right\|}_{F}^{2}}\leq \varepsilon ,\;\frac{{\left\|S^{t}-S^{t-1}\right\|}_{F}^{2}}{{\left\|S^{t}\right\|}_{F}^{2}}\leq \varepsilon$$

The proposed WNNM-based clutter removal method is summarized in Algorithm 1.


**Algorithm 1: WNNM-based clutter removal method in GPR.**
 **Input**: **X**: Raw B-scan with size *M* × *N*;    *t*_max_: Maximum number of iterations;    *ε*: Convergence error;    *λ*: Regularization parameter;       *ρ*: Weight parameter. **Output**: **S***^t^* (target response) and **L***^t^* (clutter response). **Initialize** $\;S^{0}=0;\;t_{max}=100;\;\varepsilon =10^{-3};\;\tau =10^{-15};\;t=0$ **Main iteration:   **  1. *t* = *t* + 1;  2. Calculate the singular value decomposition of X − S*^t^*^−1^, $U\Lambda V^{T}=\mathrm{svd}(X-S^{t-1})$;  3. Calculate the singular diagonal weight matrix **W***^t^*^−1^ by Equation (6);  4. Fix **S** and update **L***^t^* by  $L^{t}=U\varphi_{W}(\Lambda )V^{T}$;  5. Fix **L** and update **S***^t^* by  $S^{t}=\varphi_{\lambda }(X-L^{t})$; **Until** convergence or *t* = *t*_max_.

## 3. Experimental Results

In order to verify the effectiveness of the proposed method, both numerical simulations and real experiments are conducted. In addition, the results are compared with EMA, PCA, NMF, RPCA, and RNMF. The EMA method is compatible with the advantages of both mean subtraction and moving average methods, and is performed by subtracting the exponential average of the *N*-trace ensemble over a region of interest from each trace [8]. The peak signal-to-noise ratio (PSNR) and the improvement factor (IF) are used as evaluation criteria for quantitative analysis, which are defined as

(10) $$\;\mathrm{PSNR}\left(\mathrm{dB}\right)\;\;=10\log\frac{MN}{\sum_{i=1}^{M}\sum_{j=1}^{N}(X_{i,j}-X_{i,j}^{ref}{)}^{2}}$$

(11) $$\begin{array}{c} \mathrm{IF}\left(\mathrm{dB}\right)\;\;=10\log\left({\mathrm{SCR}}_{\mathrm{after}}/{\mathrm{SCR}}_{\mathrm{before}}\right)\;\\ \;\mathrm{SCR}=\frac{N_{C}\sum_{p\in RT}{\left|X(p)\right|}^{2}}{NT\sum_{p\in RC}{\left|X(p)\right|}^{2}} \end{array}$$

where *M* and *N* are the dimensions of the B-scan data matrix. *X* and *X^ref^* denote the clutter removal result and the reference image, respectively. In numerical simulations, the reference image is obtained by subtracting target-free data from raw data, but in real experiments it is difficult to obtain the reference image, so the IF criterion is utilized. The SCR_after_ and SCR_before_ are the signal-to-clutter ratios (SCR) of GPR data after and before applying the clutter removal method, respectively. *N_C_* and *N_T_* denote the number of pixels in the clutter region and target region, respectively. *X*(*p*) is the *p*-th pixel in the B-scan image.

### 3.1. Simulation Data Results

The simulation data are generated using the gprMax3D software [41,42,43], which can simulate real commercial antennas. Geophysical Survey Systems, Inc. (GSSI), 1.5 GHz antenna, two different targets (aluminum and plastic pipes), and six various soil types are considered, as in [26,27,28,29,30]. The detailed electromagnetic properties of the soil and the targets are listed in Table 1. The length of the aluminum and plastic pipes is 20 cm. The aluminum pipe has a radius of 2 cm and a thickness of 0.2 cm, while the plastic pipe has a radius of 2.4 cm and a thickness of 0.4 cm. The simulation domain is 1 m × 0.3 m × 0.4 m and the discretization of the model is ∆*x* = ∆*y* = ∆*z* = 2 mm. To simulate the scenario where the antennas are not parallel to the ground, we tilt the ground surface from left to right and move the antennas along a straight line. The antennas are located 2 cm above the highest point of the ground surface.

#### 3.1.1. Clutter-Removal Results in the Nonparallel Case

**Figure 1:**
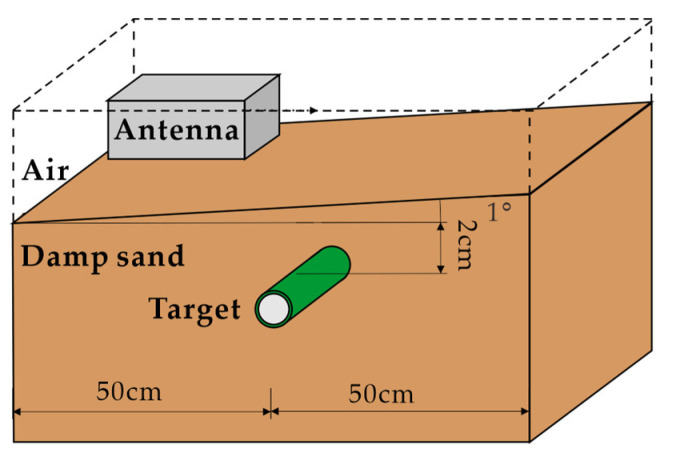
Simulation scenario of a target buried at 2 cm depth in damp sand with an angle of 1°.

To evaluate the clutter-removal performance of the proposed method in the nonparallel case, different angles between the antennas and the ground surface are considered. Figure 1 presents the simulation scenario of a target buried at a depth of 2 cm in damp sand with an angle of 1°.

In our proposed WNNM-based method, the regularization parameter *λ* and the weight parameter *ρ* have impacts on clutter-removal performance. The regularization parameter *λ* is used to adjust the proportion of sparse components and low-rank components, and a too-small value fails to remove the clutter while a too-large value weakens the targets. The parameter *ρ* determines the weight of the singular value and should be fine-tuned for different cases since a too-small value will cause the target reflection to be suppressed excessively, while a too-large value will lead to clutter suppression insufficiently. For a simple scenario with a high SCR, a larger value is recommended to ensure that more target information is retained, whereas a smaller value is suggested for a complex environment with a low SCR to successfully remove the strong clutter.

**Figure 2:**
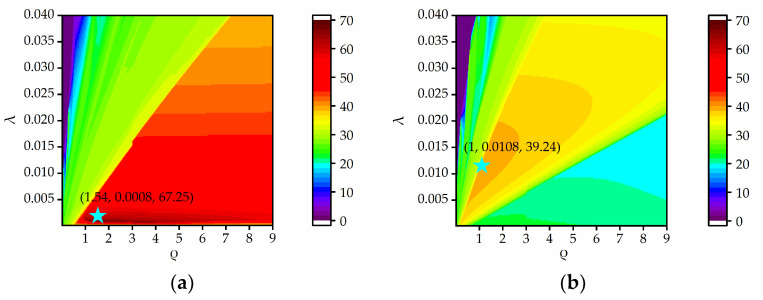
PSNR versus *λ* and *ρ*: (**a**) the angle is 0°, (**b**) the angle is 1°.

Figure 2a presents the PSNRs as a function of *λ* and *ρ* when a plastic pipe is buried at a depth of 2 cm in damp sand with a flat surface. The red area corresponds to higher PSNR values, and the blue area corresponds to lower PSNR values. A smaller *λ* and larger *ρ* can achieve larger PSNR values. The optimal parameters (*λ* = 0.0008, *ρ* = 1.54) are given by the peak (the star point) of the PSNR contour, where the highest PSNR is 67.25 dB. In the nonparallel case, as shown in Figure 2b, there is no red area since the PSNR value cannot reach above 40 dB. The orange area denotes the higher PSNR values, which correspond to a larger *λ* and smaller *ρ* compared to those in Figure 2a. The highest PSNR is 39.36 dB, and the optimal parameters are *λ* = 0.0108, *ρ* = 1. In the subsequent simulations, we select optimal parameters by maximizing the PSNR values for the WNNM, RPCA, and RNMF, and the rank *k* in the NMF and RNMF is set to 1, as in [26,27,28,29]. The sliding window in the EMA is set to 30.

**Figure 3:**
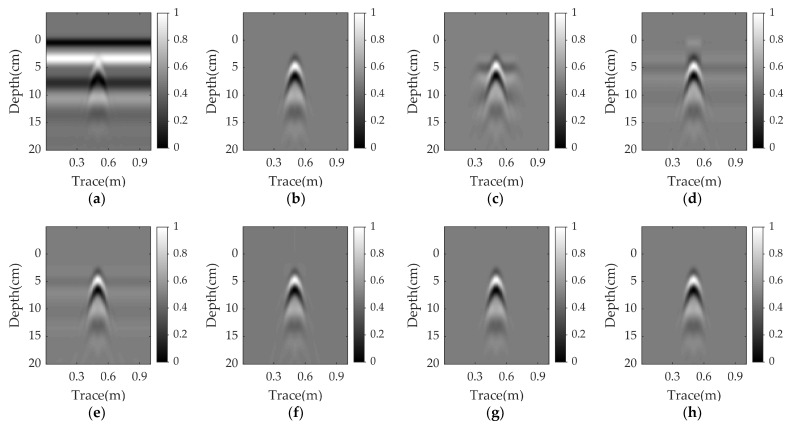
Simulation results for a plastic pipe buried at a depth of 2 cm in damp sand with a flat surface: (**a**) raw data; (**b**) reference data; (**c**) EMA; (**d**) PCA; (**e**) NMF; (**f**) RPCA; (**g**) RNMF; (**h**) WNNM.

Figure 3a shows a raw GPR B-scan image with a size of 1000 × 80. In this scenario, a plastic pipe is buried at a depth of 2 cm in damp sand with a flat surface. The corresponding clutter removal results of EMA, PCA, NMF, RPCA, RNMF, and WNNM are illustrated in Figure 3c–h. Obviously, the RPCA, RNMF, and WNNM completely remove the ground clutter and successfully extract the target reflection. Their results are similar to the reference image, as shown in Figure 3b. For PCA and NMF, the ground clutter remains, since some horizontal lines are observed and the EMA result shows some clutter around the target reflection.

**Figure 4:**
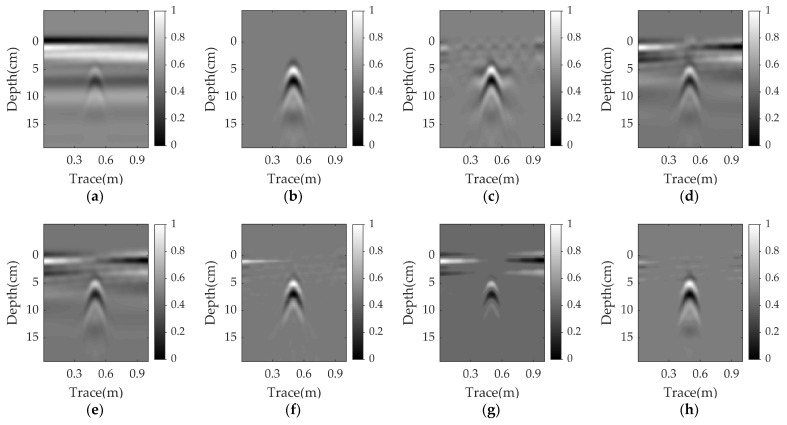
Simulation results for a plastic pipe buried in damp sand with an angle of 1°: (**a**) raw data; (**b**) reference data; (**c**) EMA; (**d**) PCA; (**e**) NMF; (**f**) RPCA; (**g**) RNMF; (**h**) WNNM.

**Figure 5:**
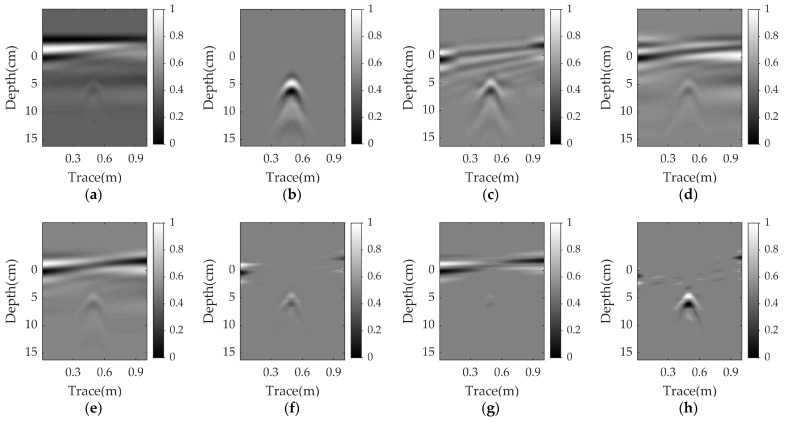
Simulation results for a plastic pipe buried in damp sand with an angle of 3°: (**a**) raw data; (**b**) reference data; (**c**) EMA; (**d**) PCA; (**e**) NMF; (**f**) RPCA; (**g**) RNMF; (**h**) WNNM.

**Figure 6:**
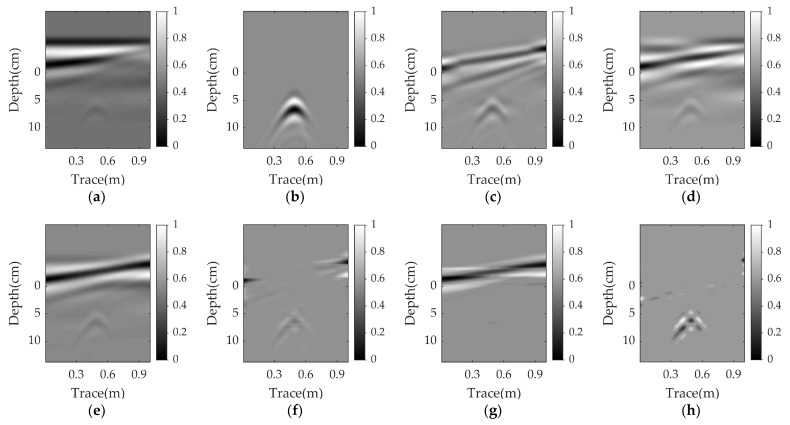
Simulation results for a plastic pipe buried in damp sand with an angle of 5°: (**a**) raw data; (**b**) reference data; (**c**) EMA; (**d**) PCA; (**e**) NMF; (**f**) RPCA; (**g**) RNMF; (**h**) WNNM.

Figure 4, Figure 5 and Figure 6 present the results for a plastic pipe buried at a depth of 2 cm in damp sand with an angle of 1°, 3°, and 5°, respectively. Clearly, the performance of all methods suffers significantly in the nonparallel case, and the greater the angle, the worse the results. In each scenario, PCA and NMF fail to remove the clutter. When the angle is 1°, the EMA achieves better results than PCA and NMF, but has more clutter compared to RPCA. RNMF removes some clutter directly above the target, but the target reflection is also suppressed. As the angle increases, the EMA cannot remove the ground clutter and the RNMF is also ineffective due to the strong remaining ground clutter and lack of target components. The proposed WNNM achieves a better visual performance by removing more ground clutter and retaining more target reflection than the RPCA. Even at a larger angle, the hyperbola reflected from the target is still obvious.

Table 2 shows the quantitative results of all methods at different angles. Since the angle is relatively small in practical applications, we set the maximum angle to 5°. When the angle is zero, the proposed WNNM has the highest PSNR, followed by the RNMF and the RPCA. These LRSD methods have higher values than EMA, PCA and NMF. When the angle is nonzero, the PSNR values dramatically decrease. The WNNM has the highest values, followed by the RPCA and EMA, which corresponds to the visual results. In some scenarios, the RNMF even has lower values than PCA and NMF, possibly due to the excessive suppression of target reflection.

To further prove the effectiveness of the proposed method in the nonparallel case, different scenarios, including different target materials, soil types, and burial depths, are considered, and the results are given in Table 3 and Table 4, where the highest PSNR values are in bold. Table 3 shows the quantitative results of an aluminum or plastic pipe buried at a depth of 2 cm in different soil types at an angle of 1°. The WNNM achieves the highest PSNR values for all kinds of soil types, followed by the RPCA and EMA, which corresponds to the visual results in Figure 4. An exception to RPCA occurs when the plastic target is buried in wet sand. In this case, RPCA has a lower PSNR value than PCA, NMF, and RNMF, since the target reflection is partially weakened while eliminating the clutter. However, under the conditions of a wet environment, the EMA result is the best method except for our method. RPCA and WNNM present the best performance in dry sand and the worst performance in wet sand. Table 4 shows the results of a target buried at different depths in damp sand with an angle of 1° using different methods. Similarly, the proposed WNNM presents the best results, about 10 dB higher than RPCA and EMA in terms of PSNR values, in most scenarios.

#### 3.1.2. Running Time

**Figure 7:**
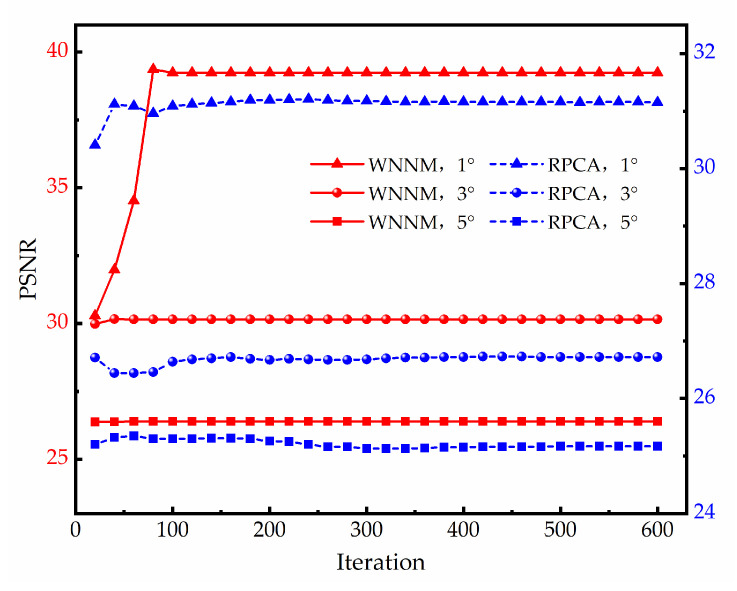
PSNR versus iteration numbers for RPCA and WNNM.

We also discuss the running time of the proposed method. The computational complexity of WNNM is *O*(*MN*^2^), which is the same as that of RPCA. However, the actual running time is also related to the number of iterations at which point the algorithm converges. Since only RPCA and WNNM are effective in nonparallel cases, we compare the convergence rates of these two methods at different angles. Figure 7 presents the PSNR versus the number of iterations. In this scenario, a plastic pipe is buried at a depth of 2 cm in damp sand. The WNNM has a faster convergence speed than the RPCA. The WNNM curves are stable after 100 iterations, whereas the RPCA converges at about 500 iterations. Table 5 presents the running time of all methods at different angles. The lowest running time is obtained by the PCA and EMA, followed by the NMF and RNMF. However, these methods are ineffective in nonparallel cases. The WNNM is about five times faster than the RPCA, which is in agreement with the results in Figure 7. Compared with the RPCA, the WNNM is more suitable for practical applications.

### 3.2. Real Data Results

In this study, two real datasets are implemented to further validate the effectiveness of the proposed method. Both visualization and quantitative results are presented for comparison. The quantitative results are based on the IF, and the parameters of EMA, RPCA, RNMF, and WNNM are selected by maximizing the IF value.

#### 3.2.1. Real Data-I

**Figure 8:**
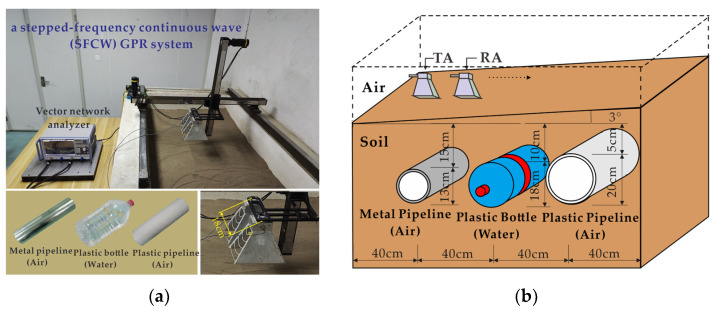
Experimental scenario Ⅰ: (**a**) Experimental setup. (**b**) Schematic diagram of the scenario.

The first real GPR data were collected with a stepped-frequency continuous wave (SFCW) GPR system developed by our research group, which includes a vector network analyzer (Rohde Schwarz ZNB40) and two broadband double-ridged horn antennas. The frequency ranged from 1.8 GHz to 5 GHz with an 8 MHz stepping frequency. The antennas with an 18 cm center-to-center distance were placed at about 5 cm above the highest point and scanned with a step size of 2 cm. Three objects (a metal pipeline, a water-filled plastic bottle, and a PVC pipeline) were buried in a dry sand tank at depths of 15 cm, 10 cm, and 5 cm, respectively. The diameters of the three objects were 13 cm, 18 cm, and 20 cm, respectively. The horizontal distance between the adjacent targets was 40 cm. The sand surface was rough, with about ±2.5 cm irregularities, and tilted from left to right. The experimental scenario is shown in Figure 8.

**Figure 9:**
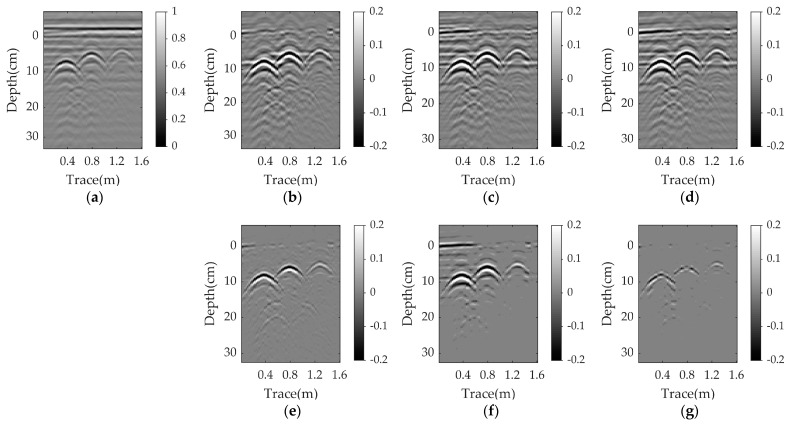
Real data-Ⅰ results at an angle of 3°: (**a**) raw data; (**b**) EMA; (**c**) PCA; (**d**) NMF; (**e**) RPCA; (**f**) RNMF; (**g**) WNNM.

**Figure 10:**
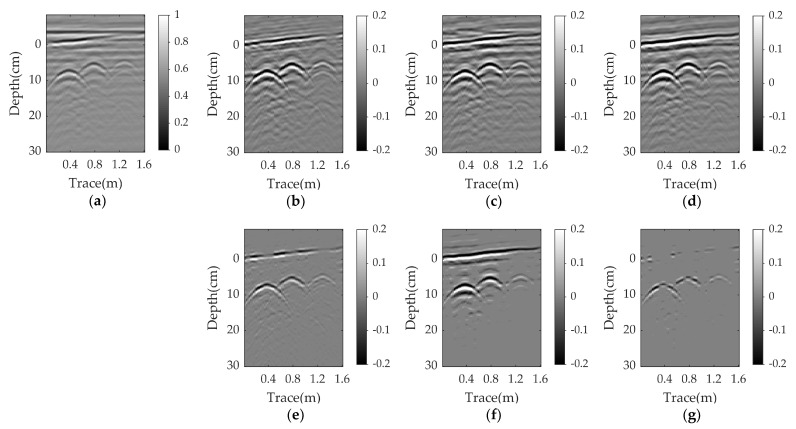
Real data-Ⅰ results at an angle of 5°: (**a**) raw data; (**b**) EMA; (**c**) PCA; (**d**) NMF; (**e**) RPCA; (**f**) RNMF; (**g**) WNNM.

Figure 9 and Figure 10 illustrate the raw data and the clutter removal results obtained by different methods when the angle is about 3° and 5°, respectively. The size of the raw data is 231 × 70. The ground reflection shows an obvious inclination, as shown in Figure 9a and Figure 10a. PCA and NMF fail to remove the clutter. The EMA and the RNMF can suppress most of the clutter at an angle of 3°, but fail at an angle of 5°. Both RPCA and WNNM successfully remove the sloping ground clutter and the background clutter. When the angle is 3°, the ground clutter is almost eliminated by the RPCA and WNNM, and the hyperbolas from the target reflections are distinct. When the angle is enlarged to 5°, the results of the RPCA and WNNM contain some clutter, but the WNNM has a clearer background and stronger target reflections than the RPCA. The corresponding quantitative results and running times of different methods are listed in Table 6. The IF values of the WNNM, as shown in bold, are significantly higher than those of other methods. Moreover, the WNNM is over 4.5 times faster than the RPCA.

#### 3.2.2. Real Data-II

**Figure 11:**
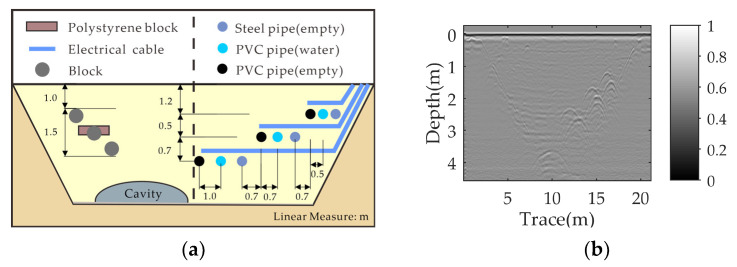
Experimental scenario Ⅱ: (**a**) Schematic view of the test site. (**b**) Raw data.

The second real GPR data come from the TU1208 open database of radargrams [44]. They were collected by a 400 MHz shielded antenna of the GSSI system in the limestone region, on acquisition line 2, at the IFSTTAR geophysical test site in France. Figure 11 presents the schematic section view of the test site and the raw GPR image. More detailed scene information can be found in [44]. The size of the B-scan data is 510 × 1418. Although the nonparallel case is not taken into account in this scenario, the field data were collected in a more complex environment than indoor experiments, so we believe they can be used to validate the effectiveness of the proposed method in practical applications.

**Figure 12:**
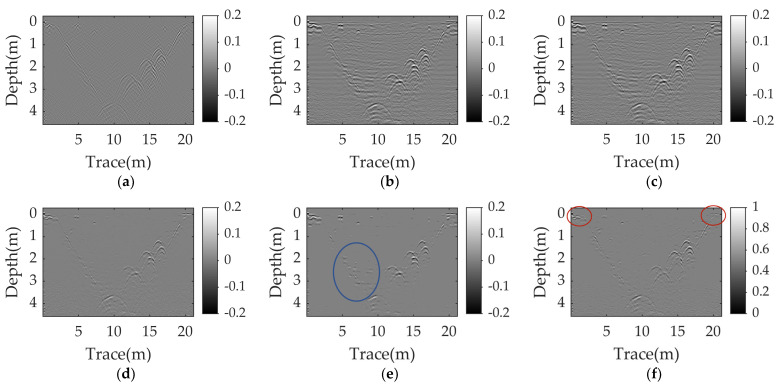
Real data-Ⅱ results: (**a**) EMA; (**b**) PCA; (**c**) NMF; (**d**) RPCA; (**e**) RNMF; (**f**) WNNM.

Figure 12 shows the clutter removal results obtained by the different methods, and the corresponding quantitative analysis is also listed in Table 6. It can be found that the ground clutter cannot be completely suppressed by all the methods. For EMA, PCA and NMF, most of the ground clutter is removed, but some background clutter is still present. The visual results of the RPCA, RNMF, and WNNM are comparable. However, the RNMF result has more background clutter, as shown in the blue circle, and more ground clutter. The WNNM performs the best in terms of removing ground clutter, as shown in the red circles. In addition, the proposed method outperforms other methods, with the highest IF value, as given in bold in Table 6, and has a slightly shorter running time than the RNMF and is roughly four times faster than the RPCA.

## 4. Conclusions

A novel GPR clutter-suppression method based on weighted nuclear norm minimization is proposed. Instead of the nuclear norm used in the traditional RPCA, it employs a non-convex weighted nuclear norm. Since different weights are assigned to different singular values, this can improve the clutter removal performance when there is an oblique angle between the antenna and the ground surface. To the best of our knowledge, few works have focused on the nonparallel case of the antenna and the ground, and existing methods are not very effective in this case, whereas our proposed method is suitable for solving this problem. Simulation and experimental results demonstrate that when the angle is zero, the proposed WNNM outperforms the popular RPCA and is comparable to the recently proposed RNMF. When the angle is nonzero, it has a better performance than the state-of-the-art methods with the highest PSNR and IF values. Moreover, it is much faster than the RPCA, which is beneficial for practical applications.

## Figures and Tables

**Table 1:** Electromagnetic properties of the materials.

Material	Dielectric Constant (F/m)	Conductivity (S/m)
Damp sand	8.0	0.01
Dry sand	3.0	0.001
Wet sand	20.0	0.1
Dry clay soil	10.0	0.01
Wet clay soil	12.0	0.01
Dry loam soil	10.0	0.001
Aluminum	3.1	2.3 × 10^7^
Plastic	3.0	0.01

**Table 2:** PSNR results for a target buried at 2 cm depth in damp sand with different angles.

	EMA	PCA	NMF	RPCA	RNMF	WNNM
**Aluminum target**						
0°	31.43	36.99	36.41	43.21	62.47	**63.21**
1°	29.53	24.87	23.40	30.46	25.57	**38.25**
2°	25.21	16.31	13.64	25.76	12.78	**26.96**
3°	24.92	16.48	15.90	28.26	15.55	**33.06**
4°	22.98	19.95	20.00	28.57	21.48	**31.91**
5°	21.59	17.94	19.14	24.87	19.92	**30.85**
**Plastic target**						
0°	32.03	34.55	36.56	38.78	60.68	**67.25**
1°	30.84	22.29	22.54	31.16	20.70	**39.24**
2°	25.63	17.83	22.20	26.73	23.32	**35.17**
3°	23.77	20.41	20.88	26.72	22.52	**30.15**
4°	23.13	19.73	19.54	22.95	20.35	**28.54**
5°	22.09	18.41	18.64	25.17	20.14	**26.39**

**Table 3:** PSNR results for a target buried in different soil types at an angle of 1°.

	EMA	PCA	NMF	RPCA	RNMF	WNNM
**Aluminum target**						
Damp sand	29.53	24.87	23.40	30.46	25.57	**38.25**
Dry sand	31.29	28.17	30.00	38.24	32.24	**41.13**
Wet sand	29.01	12.89	13.12	17.03	11.26	**32.87**
Dry clay soil	29.22	20.64	19.80	27.53	19.71	**38.79**
Wet clay soil	29.16	17.48	17.49	27.51	17.20	**38.54**
Dry loam soil	29.14	21.09	20.35	27.49	20.30	**38.64**
**Plastic target**						
Damp sand	30.84	22.29	22.54	31.16	20.70	**39.24**
Dry sand	30.03	22.57	22.57	33.00	22.04	**40.23**
Wet sand	27.57	22.72	22.94	21.26	22.01	**32.18**
Dry clay soil	30.35	22.44	22.59	31.23	20.39	**35.47**
Wet clay soil	30.40	22.70	22.73	30.17	20.66	**37.29**
Dry loam soil	30.45	22.41	22.56	31.43	20.34	**36.17**

**Table 4:** PSNR results for a target buried at different depths in damp sand with a 1° angle.

	EMA	PCA	NMF	RPCA	RNMF	WNNM
**Aluminum target**						
2 cm	29.53	24.87	23.40	30.46	25.57	**38.25**
3 cm	29.95	24.34	25.26	30.15	26.32	**41.16**
4 cm	30.22	23.25	25.24	33.89	26.53	**41.70**
5 cm	30.07	25.23	24.33	32.07	25.77	**42.18**
6 cm	29.85	24.43	23.19	31.20	24.61	**42.17**
**Plastic target**						
2 cm	30.84	22.29	22.54	31.16	20.70	**39.24**
3 cm	30.40	22.56	22.46	31.10	20.92	**38.05**
4 cm	29.82	22.45	22.26	28.26	20.87	**38.68**
5 cm	29.51	22.15	22.08	28.19	20.66	**38.27**
6 cm	29.18	21.82	21.84	29.12	20.34	**37.60**

**Table 5:** Running time (in s) for a target buried at 2 cm depth in damp sand with different angles.

	EMA	PCA	NMF	RPCA	RNMF	WNNM
1°	0.03	0.04	0.25	12.65	0.71	2.31
3°	0.03	0.04	0.26	12.13	0.70	2.23
5°	0.03	0.04	0.29	13.24	0.64	2.58

**Table 6:** IF results for real data at different angles.

		EMA		PCA		NMF		RPCA		RNMF		WNNM	
		IF (dB)	RT (S)	IF (dB)	RT (S)	IF (dB)	RT (S)	IF (dB)	RT (S)	IF (dB)	RT (S)	IF (dB)	RT (S)
Data Ⅰ	3°	12.57	0.01	6.91	0.01	7.28	0.01	16.61	0.90	12.36	0.03	**17.44**	0.19
	5°	7.62	0.01	3.85	0.02	3.89	0.01	9.11	0.91	4.47	0.02	**15.53**	0.20
Data Ⅱ	0°	6.52	0.02	6.47	0.12	6.40	3.21	7.64	65.91	8.69	18.84	**9.53**	16.23

## Data Availability

Not applicable.
